# Sars‐Cov‐2 infection and dementia risk: a nationwide, nested case‐control study

**DOI:** 10.1002/alz.090292

**Published:** 2025-01-09

**Authors:** Nika Zur, Nili Stein, Walid Saliba, Galit Weinstein

**Affiliations:** ^1^ University of Haifa, Haifa Israel; ^2^ Carmel Medical Center, Haifa Israel

## Abstract

**Background:**

Cognitive impairment following Sars‐Cov‐2 infection is common. We aimed to explore whether covid‐19 is associated with a higher risk of dementia in a nationwide sample of middle‐age and older Israeli adults.

**Method:**

This nested case‐control study was based on electronic health care data from the largest healthcare provider in Israel. From a cohort of 1,145,322 dementia‐free individuals aged ≥50y at baseline (March 2020), we matched subjects with incident dementia to controls without dementia using a density sampling by age, sex, and date of entry to the study, with a ratio of 1 to 10. The follow‐up ended in May 2022. SARS‐CoV‐2 infection was defined based on positive PCR or institutional antigen tests. Conditional logistic regression was used, adjusting for potential confounders including ethnicity, socioeconomic status, comorbidity and smoking. Interaction of covid‐19 with sociodemographic factors and comorbidities were additionally tested. Lastly, the association between covid‐19 severity and dementia were examined, with severity defined as mild without hospitalization, and hospitalization with mild, moderate and severe disease based on hospitalization discharge records.

**Result:**

Sample mean age was 77.5±9.0 years and 173,668 (57.9%) were females. Overall, 27,280 had incident dementia during the follow up, matched to 272,800 healthy controls. A positive test for covid‐19 was detected among 2,072 (7.6%) cases and 6,722(2.5%) controls. Overall, SARS‐CoV‐2 infection was associated with increased dementia risk (OR = 1.15; 95% CI 1.06‐1.25; P<0.001). There was no increased risk of dementia in those with prior mild covid‐19 who were not hospitalized compared to those without covid‐19 history (OR = 1.003; 95% CI 0.914‐1.101; P = 0.905). However, compared to no covid‐19 history, hospitalization with mild, moderate and severe disease was associated with 83%, 93% and 75% increased risk for subsequent dementia, respectively (OR = 1.833; 95% CI 1.410‐2.382; P<0.001; OR = 1.928; 95% CI 1.366‐2.722; P<0.001; OR = 1.745; 95% CI 1.316‐2.313; P<0.001). Finally, no interactions were observed.

**Conclusion:**

In this nation‐based sample, a history of SARS‐CoV‐2 infection was associated with a 15% increased risk for dementia during a 27‐months follow‐up. Increased dementia risk was observed only among covid‐19 patients who were hospitalized. Future investigations with longer follow‐ups are needed to clarify the implications of covid‐19 on brain health.

